# A Novel Application of Non-Destructive Readout Technology to Localisation Microscopy

**DOI:** 10.1038/srep42313

**Published:** 2017-02-14

**Authors:** Samuel F. H. Barnett, Mary Snape, C. Neil Hunter, Miguel A. Juárez, Ashley J. Cadby

**Affiliations:** 1 The Department of Molecular Biology and Biotechnology, Firth Court, Western Bank, Sheffield, S10 2TN, United Kingdom; 2 The Department of Physics and Astronomy, Hicks Building, Hounsfield Road, Sheffield, S3 7RH, United Kingdom; 3 School of Mathematics and Statistics, Hicks Building, Hounsfield Road, Sheffield, S3 7RH, United Kingdom

## Abstract

The fitting precision in localisation microscopy is highly dependent on the signal to noise ratio. To increase the quality of the image it is therefore important to increase the signal to noise ratio of the measurements. We present an imaging system for localisation microscopy based on non-destructive readout camera technology that can increase the signal to noise ratio of localisation based microscopy. This approach allows for much higher frame rates through subsampling a traditional camera frame. By matching the effective exposure to both the start time and duration of a single molecule we diminish the effects of read noise and temporal noise. We demonstrate the application of this novel method to localisation microscopy and show both an increase in the attainable signal to noise ratio of data collection and an increase in the number of detected events.

In recent years, several microscopy techniques have emerged which allow researchers to image biological systems at resolutions below the diffraction limit^1^. There are several distinctly different methods of achieving sub-diffraction resolutions including Structured Illumination Microscopy (SIM)^2^, Stimulated Emission Depletion (STED)^3^, Stochastic Optical Reconstruction Microscopy (STORM)^4^ and in general these techniques can be divided into two classes. One which manipulates the point spread function (PSF) of the microscope, such as SIM and STED and the other which uses prior knowledge of the system to extract more information from the PSF such as Photo-activated Localisation Microscopy (PALM)^5^ and STORM. This paper is concerned with the application of a novel imaging modality to the second and most popular class of super-resolution microscopy; localisation microscopy (LM).

The premise behind localisation techniques is to bypass the diffraction barrier by temporally separating the fluorophores so that they can be observed as single emitters. This is accomplished by taking advantage of the blinking characteristics of certain fluorophores^6^ that switch between bright (on) and dark (off) states. LM decreases the number of emitting molecules to the point where every visible molecule is isolated both spatially and temporally from all others. Each molecule can then be fitted to a function approximating the PSF to obtain a location with sub-diffraction precision^7^,^8^. Collecting many frames, each containing a stochastically different selection of emitting fluorophores, allows the reconstruction of the underlying structure at a resolution surpassing the diffraction limit.

Fitting a PSF or an approximation of a PSF to a diffraction limited signal is highly dependent on the signal-to-noise ratio (SNR) of the measurement^9^. The goodness-of-fit is dependent on the SNR and for a Gaussian PSF approximation is inversely related to the square root of the number of photons collected. Therefore, increasing the localisation precision is only achievable by increasing the signal^10^, decreasing the system noise^11^, or a combination of the two. The collection efficiency of an optical system is therefore of great importance to the amount of signal collected and as such the signal-to-noise ratio of the measurement. After the initial collection optic – generally the microscope’s objective lens – the characteristics of the camera play the major role in determining signal intensity. While other camera architectures exist, the two most commonly applied in single molecular measurements are the Electron Multiplying Charge Coupled Device (EMCCD)^12^,^13^ and the Composite Metal-Oxide Semiconductor (CMOS)^14^. Both of these camera systems have advantages and there is an ongoing debate on the most suitable technology for LM^15^,^16^.

The signal collected by a camera depends on the number of photons incident on the sensor and the quantum efficiency of the camera. For modern systems the quantum efficiency is extremely high; greater than 80% for a CMOS and over 90% for EMCCD. However, for low signal measurements such as those associated with single molecular imaging the performance of any imaging systems is limited by the system noise. There are several sources of noise and these can be specific to the camera architecture, however they can generally be described as either being time dependent or measurement dependent. For example, read noise which is caused by the work done on a pixel as the charge is emptied from the pixel occurs every time the pixel is read out. Thermal noise resulting in additional electrons in the pixel wells is time dependent; the longer the exposure time of the frame, the higher the thermal noise count.

To maximise the SNR, and therefore the localisation precision, for a single molecule experiment the frame time should match the ‘on’ time of the molecule. If the molecule begins emitting photons at the start of the frame and stops at the end of the frame, all the photons collected occur in a single frame, the image suffers read out noise, but only once and any time dependent noise is minimised. However, if the molecule starts its emission cycle towards the end of a frame and continues emitting into a second frame, then the number of photons collected is shared between two frames. This splits the signal and doubles the read noise for the event; the time dependent noise is also increased because the event now lasts two frames. This is shown graphically in Fig. 1. As molecules do not emit for a set time, matching the exposure for all events is impossible.

If the read noise could be removed altogether from the system, then one could imagine running the camera with exposures much shorter than the average ‘on’ time of a molecule. Although the signal would be split over many frames, the frames in which the event occurs could be summed together. This would minimise the amount of time dependent noise whilst maximising the signal.

## Non-Destructive Readout Cameras

In EMCCD and CMOS devices images are formed when light is collected by the camera in discrete wells known as pixels. Each pixel is active for a given amount of time, the exposure time, after which the pixel is read by removing electrons from the wells and recording the number of electrons collected. In some CMOS designs it is possible to measure the amount of charge accumulated without reading out the pixel, i.e. without removing the electrons from the well^17^. This essentially allows the sub-sampling of frames, where each sub-sample is a measurement of the amount of charge in the pixel at a particular time, this value increases until the pixel is emptied.

**Figure 1:**
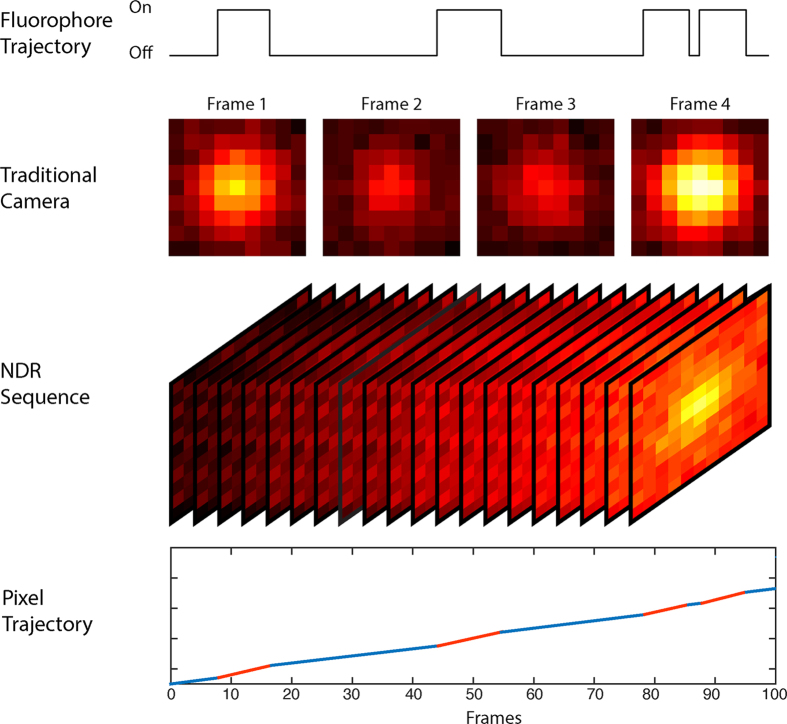
Comparison of a traditional camera to an NDR camera. In a traditional system each frame is independent from the previous frames. The top trace in the figure shows a single molecule emitting light in discrete time periods. The stochastic nature of these events leads to the signal being split when there is a mismatch between the frame start and the molecule start, this is shown in the traditional camera depiction of frames 2 and 3. Additionally, temporally separate events can be combined into one when in a diffraction limited area as in frame 4. In the NDR sequence the amount of charge in each pixel is continuously measured but the electrons collected are not read out leading to an increasing gradient (from both noise and signal). The pixel trajectory shows the increase in signal for an NDR pixel is made up of contributions from thermal noise (blue) and photons collected from a molecular event (red). The gradient of these is different allowing the start and end times of the molecular emission to be calculated.

As previously mentioned, the signal-to-noise characteristics of a localisation based measurement map directly to the spatial resolution of the microscope^9^. To improve the resolution one must either increase the amount of signal collected, i.e. the number of photons, or reduce the amount of noise within the imaging system. As localisation techniques move from using dye based probes to protein based probes with generally lower photon yields^18^, the need to control noise within the measurement system becomes increasingly important. Due to the resolution of each measurement being dependent on the collection of light from a single molecule, it is useful to move away from the idea of an image frame and instead deal with the molecules separately. Each time a molecule enters the ‘on’ state and emits light that is collected by the microscope we have a collection event. Currently events from multiple molecules are grouped together into single frames, so the start and end of the individual events do not in general coincide with the start and end of a single frame, see the traditional camera timeline in Fig. 1.

Ideally the start of the imaging frame would occur at the beginning of a single event and finish at the end of that event. This maximises the number of photons collected and minimises any read noise and temporal noise associated with the camera measurement.

In this work we introduce a third imaging modality based on non-destructive readout (NDR) camera technology. In an NDR camera the amount of charge in each pixel is read repeatedly without the charge being emptied, this means that each measurement and therefore the image, is the sum of the previous value plus noise and newly collected photons, see the NDR sequence in Fig. 1. In the past this had limited the usefulness of NDR cameras to studying static systems as motion blurred the image^19^. However, in a STORM/PALM experiment the underlying image typically does not move so there is no motion blur associated with the images, but there are time dependent events in the frames, i.e. the molecular blinking. NDR mode is therefore suited to LM as the events are either ‘on’ or ‘off’ and yet do not move in space.

As pixels have a finite depth the wells must at some point be emptied of electrons, this is performed with a clean-out frame in which the camera acts as a normal CMOS sensor. The number of NDR frames between cleaning frames can be defined before the experiment and should be frequent enough to stop the wells becoming saturated. In this work, cleanout frames were set to occur at every 100 NDR frames. However, this can be altered for samples with different signal intensities. To allow comparison with standard CMOS cameras the frame rate of the camera used in these measurements was 2500 NDR fps, with a clean-out frame rate of 25 fps.

Based on the similar concept of increasing the temporal resolution of LM, a novel use of single photon avalanche diodes (SPADs) for localisation microscopy has previously been demonstrated^20^. This allowed the collection of datasets at a much higher frame rate with lower read noise than is typically attainable with current systems. However due to the binary nature and low-fill factor of the pixels in a SPAD array the achievable resolution is lower than that with a traditional imaging setup.

To improve the SNR of LM we have developed an imaging system which allows us to sub-sample a normal CMOS frame with respect to time using NDR. For this study we used a Da Vinci 2 K CMOS based imaging chip running at 2500 fps. The sensor is mounted in a camera that was generously on loan from SciMeasure^21^. The camera is built on a CMOS architecture and can be operated in a non-destructive readout mode (NDR). When running in NDR mode the camera records the amount of charge accumulated in each pixel without removing the photoelectrons; this does not incur read noise. The DaVinci cameras have a Quantum Efficiency of 65% at 600 nm and can achieve frame rates as high as 14 K fps when windowed down sensors are used.

## Method

To investigate the application of NDR cameras to STORM we imaged a sample of fluorescently labelled *Staphylococcus aureus*. An overnight culture of *Staphylococcus aureus* (SH1000) containing a single colony in 10 ml BHI (Brain Heart Infusion 37 g l^−1^) was prepared and grown at 37 °C, 250 rpm shaking for 18 h. This was re-cultured to an OD600 of 0.05 into 50 ml BHI (same growth conditions as Overnight) & left to grow to reach mid exponential phase (OD600~0.3). The sample was spun down for 5 minutes at 5,000 g and the supernatant removed. The sample was fixed for 30 minutes in 8% (w/v) formaldehyde. Once fixed, the sample was labelled with Alexa Fluor 647 (Invitrogen). The sample was finally washed in PBS and diluted to an appropriate concentration for microscopy (~100 μl) before mounting. Cells were imaged on a Nikon TiE inverted optical microscope using a 647 nm laser at a power density of 3 kW/cm^2^. A standard Cy5 Nikon filter set (590–650 nm excitation, 660 dichroic, 663–738 nm emission) was used for imaging. Images were collected using the DaVinci camera chip at 2500 NDR fps (0.4 ms exposure) with every 100th frame being used to clear out the pixel wells of the camera. The point spread function of the microscope was expanded over 3 × 3 pixels on to the camera allowing for localisation measurements to be performed on single molecules.

The image data was processed with custom MATLAB scripts where the frames were divided into groups known as blocks with each block occurring between two clean-out frames. The length of these blocks was either 100 frames long or 500 frames long depending on the sample conditions with brighter samples requiring more regular clean-out frames. A block can be considered the equal of a normal CMOS frame, we in general use 100 NDR frames to a block, keeping the block length comparable to a normal CMOS experiment. To normalise the pixel offsets across the camera for each block the first image is subtracted from each frame in the block. This has the effect of acting as a correlated double sampling (CDS) measurement which is a common practice in CMOS imagers for reducing the noise^22^.

In conventional CMOS cameras, an offset map is applied to correct for variations across the CMOS chip. However, it is not possible to get an offset value for each pixel during imaging, thus an average offset for each pixel is used to approximate for this. NDR has the advantage that it can measure the offset for each pixel at every clean out frame before light is collected by the camera rather than having to use an average.

Due to noise within the optical system the number of electrons in an NDR pixel is constantly increasing with time. Collection of photons from a single molecular event causes an increase in the number of photons collected per unit time. Therefore, single molecular events were identified by a positive change in the gradient of a pixel’s value over time. To find candidate events in each block the images were first Gaussian filtered to remove peaks due to noise. The standard deviation of each pixel was calculated over every 10 frames (equivalent to 4 ms). Higher values in the standard deviation map indicated possible single molecule events within those 10 frames. To further filter the standard deviation map a difference of Gaussians filter was applied, which highlighted regions of high standard deviation with a spatial extent similar to the point spread function of the microscope. To this final map a threshold was used to select potential events.

**Figure 2:**
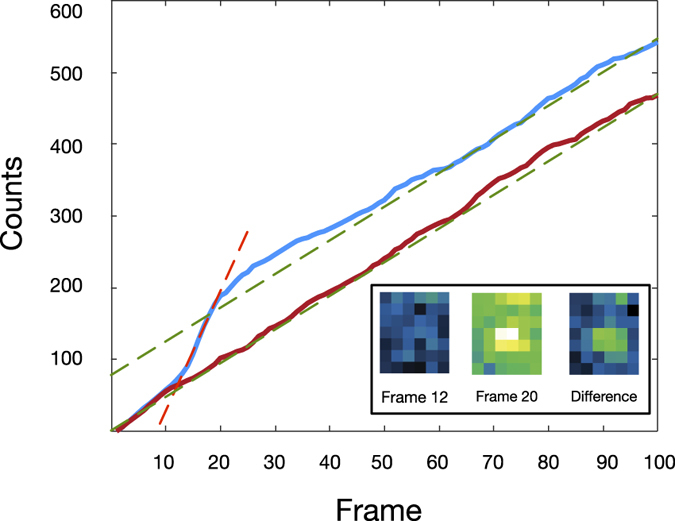
The intensity of two pixels between two clean out frames in an NDR block. The red line shows a pixel which does not detect a molecular event. The increase in intensity in this pixel is due to thermal noise within the camera, the gradient due to thermal noise is given as a green dashed line. The blue line represents the time trace for a pixel which detects a molecular event, the event occurs between frames 12 and 20 which represents a time difference of 3.2 ms. The increase in gradient between those frames is due to collected photons emitted by the molecule, this gradient is shown as a dashed orange line. The inset shows the event represented by the blue line for frames 12 and 20 and the difference between these frames.

Once a potential candidate pixel had been identified the values of the pixel and its cardinal pixels were collected throughout the entire block, effectively representing the history of the event. A typical pixel history containing an event is given in Fig. 2. The figure shows two pixel traces; one in which an event occurs is given in blue and one without an event is given in red. The two traces differ between frames 12 and 20, the increase in the gradient is due to extra photons being collected by the pixel due to a molecular event, the event lasts for 3.2 ms. This is highlighted by the dotted orange line. The camera used in this work was not cooled and so showed significant thermal noise, giving rise to a constant increase in the counts. The gradient due to thermal noise is given as a dashed green line, this gradient was constant across a single experimental run.

To find an event in the time trace the data was differentiated and then de-noised with a Chung-Kennedy filter^23^; regions of high variation represent a molecular event. The start and end frame were calculated using an intensity threshold and only events lasting longer than 5 frames (2 ms) were selected to reduce noise spikes being classed as an event. To optimize the signal to noise ratio for a single event only NDR frames which contain photons obtained from a molecular event should be collected, thus maximizing the signal while minimizing the noise. As such the overall event is then calculated by taking the difference between the first frame and the last, this would be the equivalent of subtracting frame 12 from 20 in Fig. 2, demonstrated as in inset in the figure. This process is repeated for all the candidate pixels in a single block, then the next block is processed.

Once all the events had been extracted and stored, the molecular locations were then found by least-squares fitting of the extracted events to a two-dimensional Gaussian effectively giving sub-pixel resolution. The positions of the events are then plotted using Thunderstorm^24^ to form an image.

To retrieve regular CMOS images from the NDR data the difference between the first and final frames of a block was taken. This CMOS equivalent data was then processed using Thunderstorm to locate events. The SNR of these events was calculated in the same manner as for the NDR data.

## Results and Discussion

**Figure 3:**
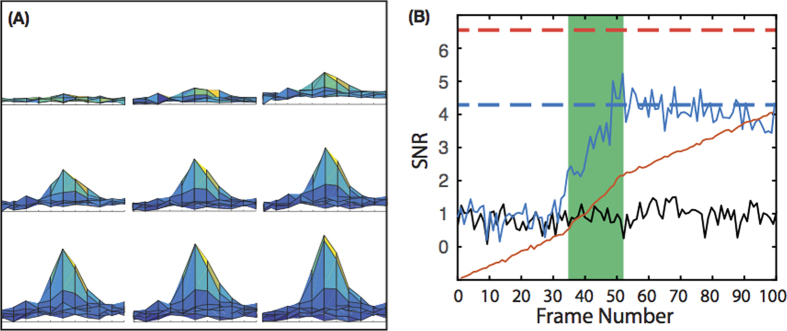
(**A**) A typical event captured over 40 NDR frames, each image is 5 frames (2 ms) apart, the SNR increases over time. (**B**) The SNR (solid-blue) of a single event occurring in a block. During the first 33 frames the SNR is close to 1 until the molecule begins emitting photons and the SNR increases to 5. When emission ceases the SNR decreases as there are no more signal photons collected but because the noise is time dependent it increases. Shown below the SNR is the pixel trace for the event (solid-red), showing the increase in gradient occurs simultaneously with the increase in SNR. The dashed blue line represents the SNR value for the frame. The dashed red line represents the maximum possible SNR for the event by subtracting the frame at the start of the molecular event from the final frame of the molecular event (highlighted in green). The solid black line shows the SNR, over 100 NDR frames, for a single pixel which has been masked so that no light falls on the pixel.

When windowed down to 2048 × 180 the NDR camera runs at 2500 fps, roughly 100 times faster than a normal CMOS camera with the NDR not incurring any additional read noise penalty for the higher frame rate. This allows sub-sampling of CMOS frames giving much higher temporal resolution. By calculating the difference between the NDR frame at the start and end of an event it is possible to tailor the exposure for each molecular event and optimise the SNR. A typical ‘on’ event captured by the NDR camera is given in Fig. 3A. The images in Fig. 3A were created by taking the difference between successive NDR frames and the first frame of the event, i.e when the molecule is first detected. There is a difference of five NDR frames between each image in the figure which shows the increase in signal-to-noise over time. The maximum SNR is achieved by taking the difference between the frame prior to the start of the event and the last frame in which it emits. It can be seen that the SNR increases with successive frames while the molecule is emitting. Figure 3B shows the SNR calculated for a single event over one hundred NDR frames which corresponds to a single CMOS frame. The SNR is given as a blue solid line and the normalised intensity of the main pixel is given as a red solid line. Over the first 33 frames the SNR oscillates around 1.0 and is non-significant for an event. The SNR then rapidly increases over the period highlighted in green which is the ‘on’ period of the molecule. Once the emission stops the SNR drops as the effects of temporal noise begin to dominate. The one hundred NDR frames are equivalent to a single CMOS frame, for this event the final SNR for a CMOS measurement would be 4.2, shown as a blue dashed line. However, the NDR allows the measurement of the signal and noise for only the time period the molecule is emitting (highlighted in green) i.e NDR frames 30 to 50, this gives a SNR of 6.5 and is shown as a red dashed line.

To further investigate the increase in SNR, we imaged several *Staphylococcus aureus* cells labelled using the standard STORM dye Alexa Fluor 647 and calculated the SNR for all the events found in a single imaging experiment. The SNR was calculated by measuring the peak intensity of the events and the mean of the pixels surrounding the event at a distance greater than the PSF. To compare data collected using NDR mode to data which does not take advantage of the NDR capabilities of the camera we took the difference between the first frame after a clean-out frame and the last frame before the next clean-out frame, this simulates the camera running as a normal CMOS with a frame rate of 25 fps. The frames between sequential clean-out frames we define as a blocks.

**Figure 4:**
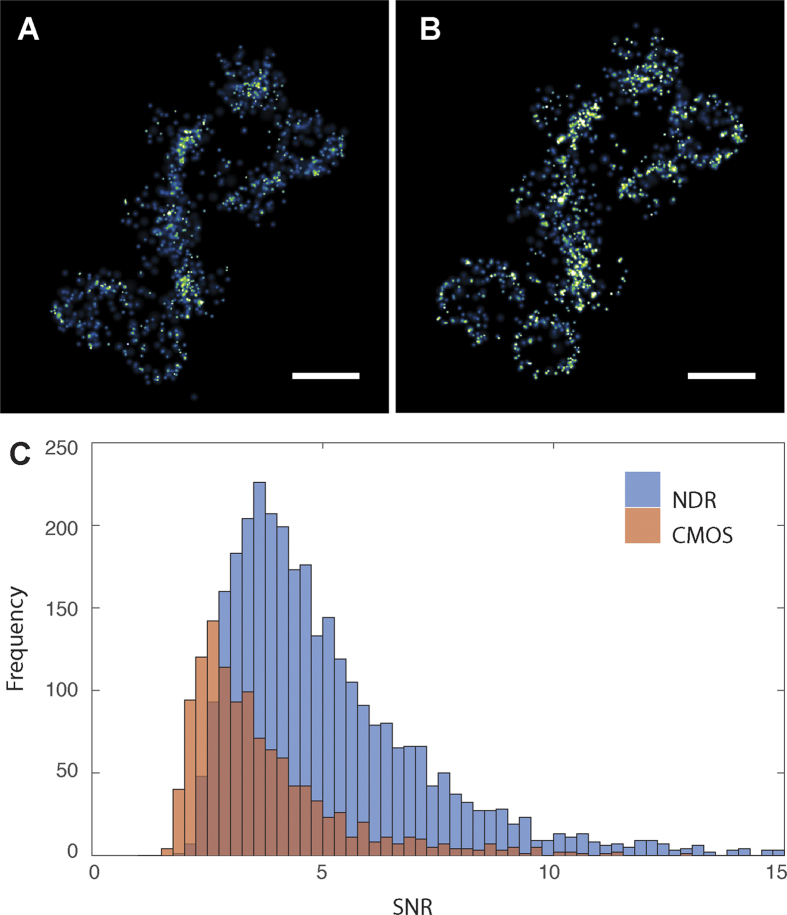
Comparison of reconstructions of pseudo-CMOS data and NDR data. (**A**) CMOS data, created by subtracting the final NDR frame in a block from the first, equivalent to a 25 fps CMOS image. Data were reconstructed in ThunderSTORM. (**B**) Events from the NDR data were extracted using custom MATLAB scripts before localisation was performed by fitting to a Gaussian function. Both images are scaled to have the same dynamic range and the scale bar is 1 µm. (**C**) The SNR values for all the localisations in NDR (blue) and CMOS (red).

The images given in Fig. 4A and B show the reconstructions using CMOS data and NDR data respectively. The CMOS equivalent data for each block was saved as a tiff and processed using Thunderstorm^24^ to locate events, while for the NDR data localisation was performed using custom software available in the supplementary materials. Both images show the same collection of cells with the data processed using the NDR method having a larger number of events and therefore giving a higher quality image. The histograms of the SNR for the 3000 NDR events and 1000 CMOS equivalent events used to construct images 4A and 4B are given in Fig. 4C. The mean SNR is higher for the NDR data and shows a significant tail towards higher values. The reduced number of events in the CMOS equivalent data is due to the inability to detect short lifetime events and events that are spatially and temporally close. The signal to noise ratio and therefore the location precision can be increased by selecting only frames which contain photons collected while a molecule is emitting light. This is not possible with traditional imaging modes because every frame incurs a read noise penalty.

Furthermore, the increased time resolution allows the ‘on’ time of single molecules to be measured with a higher degree of precision. This can be seen by taking a histogram of the molecules’ ‘on’ times which is given in supplemental information Fig. 1. The emission period for a set of single molecules follows the expected Poisson distribution^25^. The stochastic nature of the ‘on’ times and Poisson nature of the durations makes it impossible to select a frame time that is ideal for every event using a traditional sensor, while the NDR approach allows each molecular event to have their own frame.

Finally, the increased temporal resolution can be used to isolate events which are spatially and temporally close. Supplementary information Fig. 2 shows the time trace of an event occurring in a single CMOS frame. The event is shown in SI Fig. 2 part D and the differentiated time trace of the center pixel and the 4 cardinal pixels is given in SI Fig. 2 part A. It can be seen from the time trace that the pixels identified with green crosses are associated with an event which happens between NDR frames 50 and 70, while the pixels identified with blue crosses also belong to a secondary event occurring between frames 10 and 30. By selecting frames 10−>30 and 50−>70, it is possible to separate out the two events and these are given in SI Fig. 3 parts B and C. The two events shown in B and C would be misidentified as a single molecule in a typical CMOS measurement.

LM has a very low temporal resolution with a typical image being acquired on a timescale of minutes. With such long collection periods the distance that the microscope can drift is increased; lowering the accuracy of the resulting reconstruction. This is typically corrected either through the use of fiducial markers that are used to track the drift and then realign the data back to the original coordinate system or via cross-correlation^26^. The high frame rate of this camera means that data can be acquired much more rapidly and thus the levels of drift are decreased.

## Conclusions

We have used NDR camera technology to perform LM, specifically STORM. NDR provides sub-frame resolution which affords many advantages over conventional imaging techniques. The first advantage is the ability to tailor exposure times to individual single molecule events. This greatly increases the mean SNR which should translate directly in to increased localisation precision. A second advantage originating from the increased temporal resolution is a higher event detection rate. In a typical data set of bacterial cells, shown in Fig. 3 the number of events detected rises from 1000 to over 3000. Finally, the high temporal resolution of the NDR system allows us to resolve events which would overlap in a single CMOS frame. The results in this work were taken with a non-optimal camera set up, however, we believe that with simple improvements to the hardware that NDR technology shows great promise for LM.

## Additional Information

**How to cite this article**: Barnett, S. F. H. *et al*. A Novel Application of Non-Destructive Readout Technology to Localisation Microscopy. *Sci. Rep.* **7**, 42313; doi: 10.1038/srep42313 (2017).

**Publisher's note:** Springer Nature remains neutral with regard to jurisdictional claims in published maps and institutional affiliations.

## Supplementary Material

Supplementary Information
