# Supplementary material for: A Novel Application of Non-Destructive Readout Technology to Localisation Microscopy
Source: Sci Rep. 2017 Feb 14;7:42313. doi: 10.1038/srep42313 (PMC5307961; doi:10.1038/srep42313)
Supplement: Supplementary Information [file srep42313-s1.doc]

**A Novel Application of Non-Destructive Readout Technology to Localisation Microscopy**

Samuel F. H. Barnett^1^, Mary Snape^2^, C. Neil Hunter^1^, Miguel A. Juárez^3^,

Ashley J. Cadby^2*^

^1^The Department of Molecular Biology and Biotechnology, Firth Court, Western Bank, Sheffield, S10 2TN, United Kingdom.

^2^The Department of Physics and Astronomy, Hicks Building, Hounsfield Road, Sheffield, S3 7RH,
United Kingdom.

^3^School of Mathematics and Statistics, Hicks Building, Hounsfield Road, Sheffield, S3 7RH,
United Kingdom.

| **Parameter** | **Da Vinci 2K** | **ZYLA 5.5 sCMOS** |
| --- | --- | --- |
| **Type** | Non-Pinned Photodiode CMOS | Pinned Photodiode  CMOS |
| **Imaging Array** | 2048 x 2048 | 2048 x 2048 |
| **Pixel size** | 15 x 15 um^2^ | 6.5 x 6.5 um^2^ |
| **Micro-lens** | NO | YES |
| **Non-Destructive readout (DNR)** | YES | NO |
| **Max FR @ full frame Non-CDS** | 228 fps | NA |
| **Full Well** | 18,000e- | 30,000e- |
| **NON NDR mode read noise** | 2.8e- | 1.9e- |
| **QE** | 65% @ 600nm | <80% @ 600nm |
| **Dark current -10°C** | 1.6e- /pixel/sec | 0.5e- /pixel/sec |

**Table 1.** **The above table gives an overview of the some of the key characteristics of the Da Vinci camera and a standard sCMOS camera for comparison.  The dark current is higher for the Da Vinci Camera as currently the camera is not cooled.  In non-NDR mode, i.e. as the camera operating in standard CMOS the read noise varies depending on the frame rate.  Fixed pattern noise is greatly reduced in NDR cameras as the initial frame after the clean out frame is used as a dynamic correction.  When operating in NDR mode the camera does not incur read noise as the electrons are not removed from the well.**

During NDR readout the camera measures the voltage across each pixel to determine the charge stored with in the well. The amplifier measuring voltage across the pixel has a gain of 1.2 and measurement error of 8.5 nV /sqrt(Hz).  This signal is passed through a second amplifier with of a gain of 1.2 and a measurement error of 6 nV /sqrt(Hz).  Therefore, the output of these amplifiers is 134 µV per electron.  The total noise for the electrical measurement is 10.4 nV/sqrt(Hz), giving a total amplifier noise of 86 µV at a pixel frequency of 68MHz.  However, it should be noted that frames from NDR are positively correlated, implying that the standard deviation of the difference between any two (in the same run) will be smaller than 1.41 times the standard deviation of a single frame.

In conventional CMOS one takes the difference between the frame of interest and a reference frame.  If these two are independent, the variance of the difference is the sum of the variances from each frame; if both have the same variance you will get the sqrt(2) factor. With the NDR, we know that frames are not independent and indeed have a positive correlation (the camera is adding something each NDR frame), this implies that the variance of the difference between two frames is the sum of their variances minus the covariance, which is positive and thus smaller than in CMOS.

**Figure 1 supplementary materials: Histogram of the calculated on times of the events in figure 3B main text. An exponential distribution with rate 0.2 (0.195) was fitted to the data, yielding a mean duration of 5.12ms. The exponential fit is consistent with the number of events following a Poisson law with a similar rate. Note that the fit at shorter on times is not as good as we are censoring the data for events greater than 6 frames.**


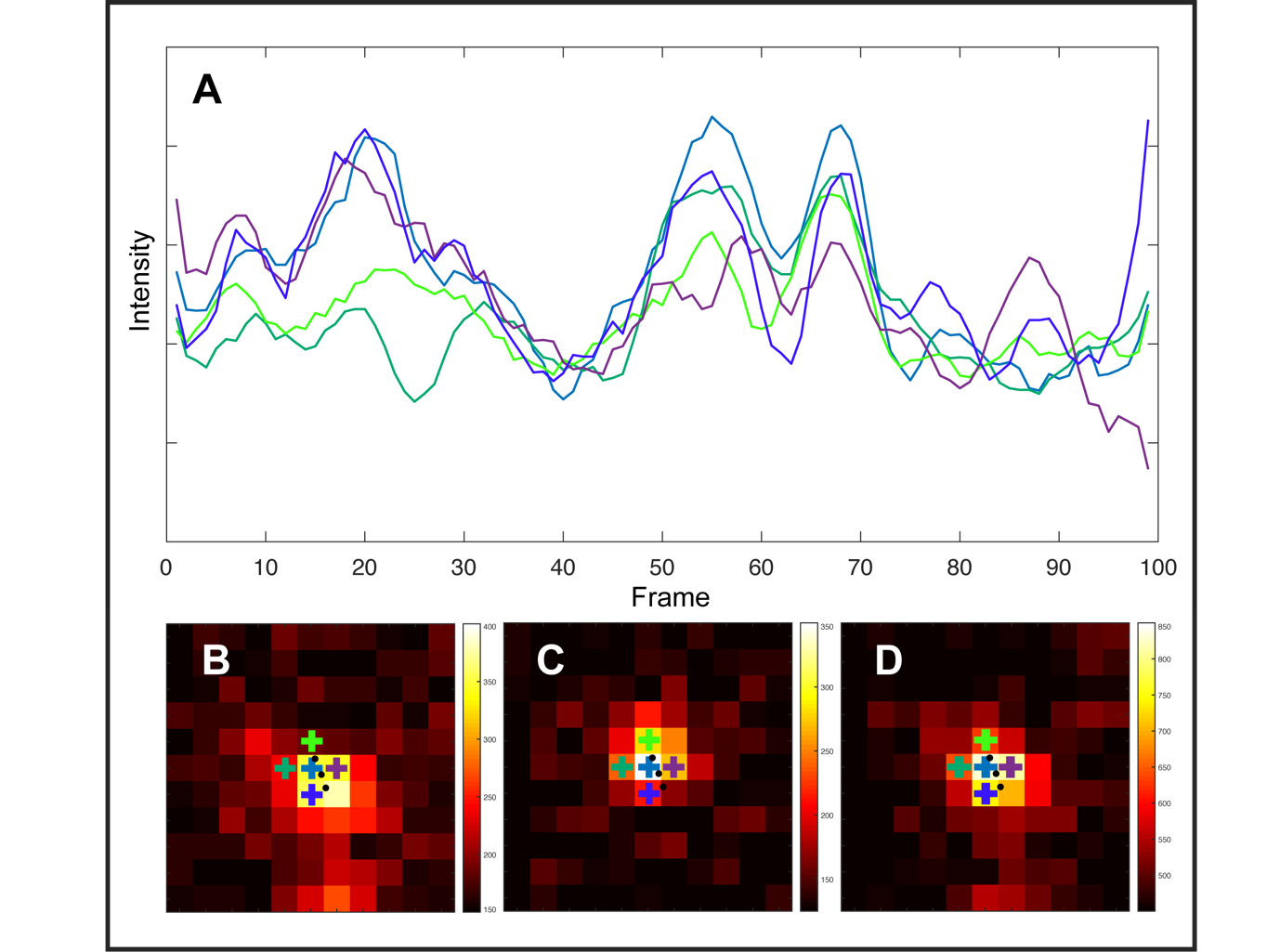


**Figure 2 supplementary materials: Part A shows the time-traces of the 5 central pixels which are used to identify a single molecule event. As can be seen between frames 10 and 40 two of the traces have a low response whereas between 70 and 40 all five traces show a response. This indicates that these two periods are the result of two separate molecules. This is further demonstrated in B and C which show the first and second events respectively. D shows the CMOS equivalent frame which lasts the entire time period and falsely appears as a single event**.

Figure 2, shows a single CMOS event, using the NDR capabilities of the camera this event was seen to be two events separated in time. We have calculated two events measured by NDR to be 170 nm ± 50 nm apart. The CMOS measurement measured a single event located in between these two events.


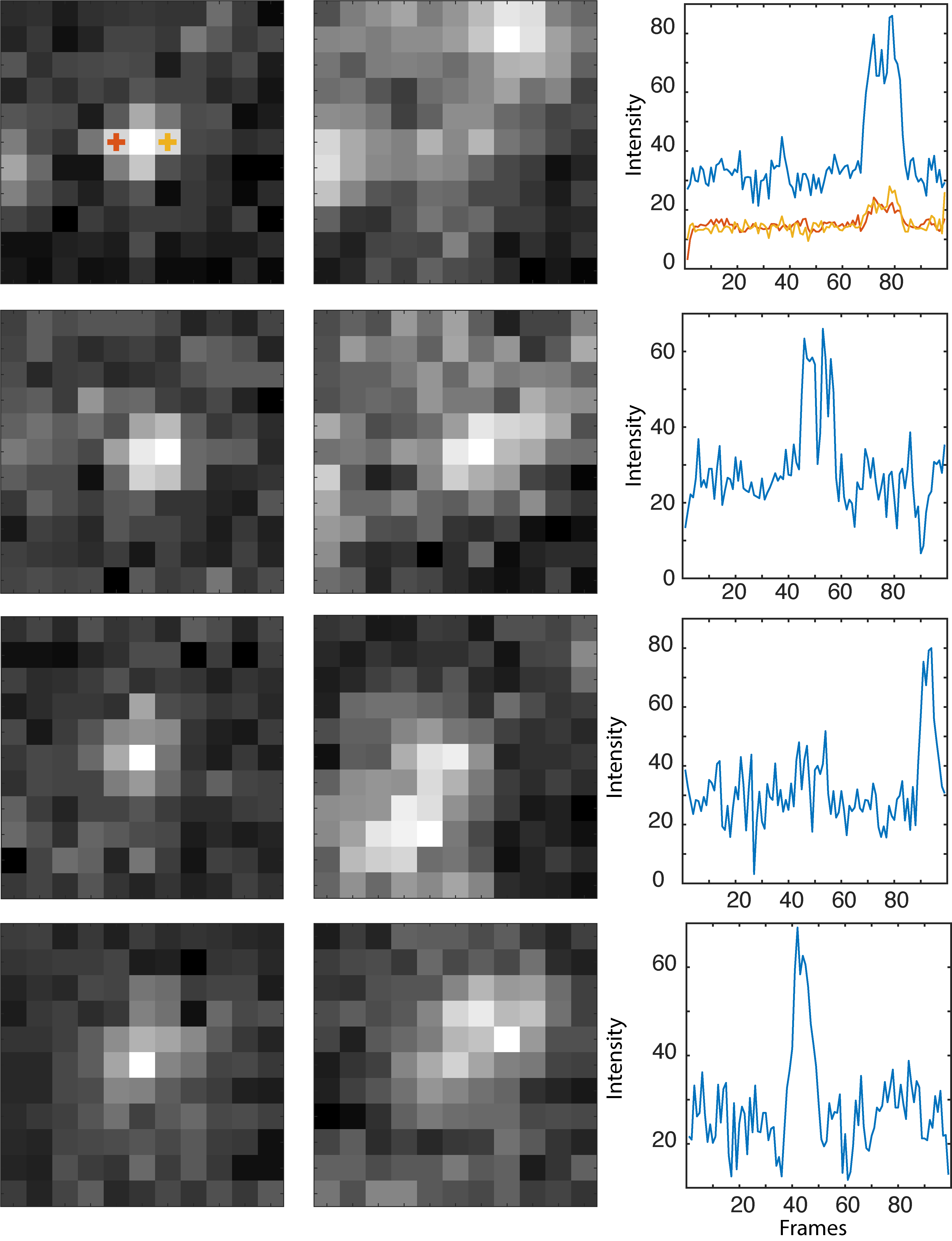


**Figure 3 supplementary materials:. The code we use for detection requires a number (5, linked to the size of the point spread function) of adjacent pixels to have high values for classification as an event.  Furthermore, single pixel events would be filtered out from our localization code as they are too narrow, width filtering is a standard method in STORM microcopy for the rejection of noise.  To emphasize this, we show the time trace of several events showing the NDR and sCMOS frames for the same event.   The NDR events are clearly shown and the pixels surrounding the central pixel indicate an event as is expected for a single molecule which in our system is spreads over 3x3 pixels due to the diffraction limit.  The first image of the figure shows the time trace for the sum of the event and the horizontal cardinal pixels, all show the same temporal response.**


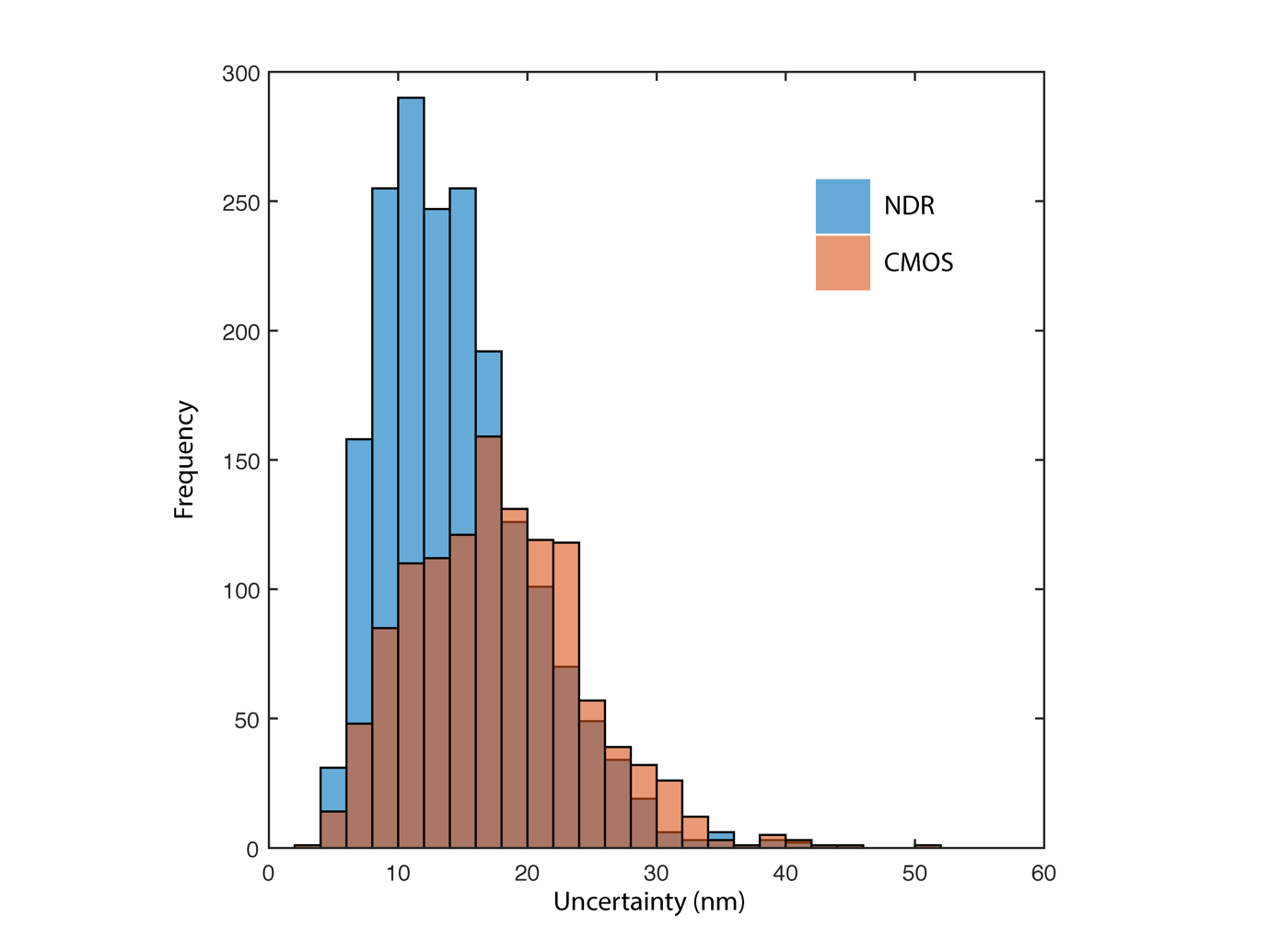


**Figure 4 supplementary materials: The histogram show the localisation precession, for the detected events in figure 3 from the main text, as calculated by ThunderSTORM^3^. The CMOS data was passed directly to ThunderSTORM. For the NDR data each event was extracted using the NDR method described in the main text, once extracted ThunderSTORM was used to calculate the precision of the localisation fit for all events. We also used Fourier ring correlation (FRC) to investigate the resolution of the final image, FRC measurements performed on the images show an improvement for NDR.  However, due to limited collection time we are most likely operating in the low-density regime for both measurements.**


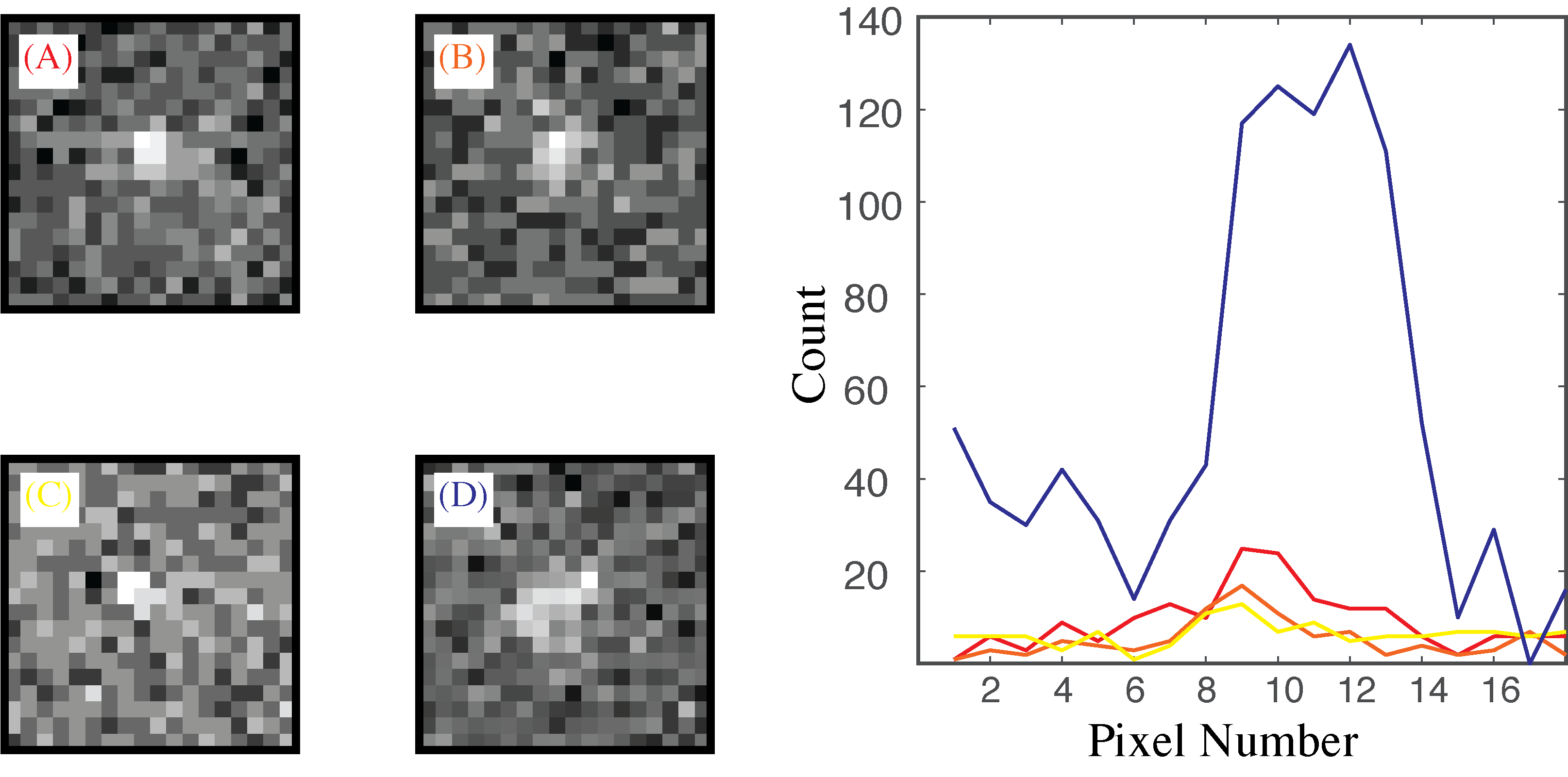


**Figure 5 supplementary materials: Three single molecule events of Alexa Fluor 647 nm captured on an Andor Zyla CMOS camera at various frame rates 100 fps (A), 200 fps (B) and 500 fps (C). Image (D) shows a single molecule event captured using the NDR camera, the image is the difference between 5 NDR frames which is the equivalent of 500 fps, as the NDR camera runs at 2.5K fps. The line traces through the resulting events are shown in the plot to the right, with the CMOS data being red (100 fps), orange (200 fps) and yellow (500fps), the plot of the NDR data is given in blue. The imaging conditions for the CMOS and the NDR images are identical except for the effective pixel size which was 150nm in the NDR and 108 nm in the CMOS.**
